# Gingiva Equivalents Secrete Negligible Amounts of Key Chemokines Involved in Langerhans Cell Migration Compared to Skin Equivalents

**DOI:** 10.1155/2015/627125

**Published:** 2015-10-11

**Authors:** Ilona J. Kosten, Jeroen K. Buskermolen, Sander W. Spiekstra, Tanja D. de Gruijl, Susan Gibbs

**Affiliations:** ^1^Department of Dermatology, VU University Medical Center, De Boellelaan 1118, 1081 HV Amsterdam, Netherlands; ^2^Department of Oral Cell Biology, Academic Center for Dentistry (ACTA), University of Amsterdam and VU University, Amsterdam, Netherlands; ^3^Department of Medical Oncology, VU University Medical Center, De Boellelaan 1118, 1081 HV Amsterdam, Netherlands

## Abstract

Both oral mucosa and skin have the capacity to maintain immune homeostasis or regulate immune responses upon environmental assault. Whereas much is known about key innate immune events in skin, little is known about oral mucosa. Comparative studies are limited due to the scarce supply of oral mucosa for ex vivo studies. Therefore, we used organotypic tissue equivalents (reconstructed epithelium on fibroblast-populated collagen hydrogel) to study cross talk between cells. Oral mucosa and skin equivalents were compared regarding secretion of cytokines and chemokines involved in LC migration and general inflammation. Basal secretion, representative of homeostasis, and also secretion after stimulation with TNF*α*, an allergen (cinnamaldehyde), or an irritant (SDS) were assessed. We found that proinflammatory IL-18 and chemokines CCL2, CCL20, and CXCL12, all involved in LC migration, were predominantly secreted by skin as compared to gingiva. Furthermore, CCL27 was predominantly secreted by skin whereas CCL28 was predominantly secreted by gingiva. In contrast, general inflammatory cytokines IL-6 and CXCL8 were secreted similarly by skin and gingiva. These results indicate that the cytokines and chemokines triggering innate immunity and LC migration are different in skin and gingiva. This differential regulation should be figured into novel therapy or vaccination strategies in the context of skin versus mucosa.

## 1. Introduction

Both oral mucosa and skin have the capacity to maintain immune homeostasis and regulate immune responses upon environmental assault. They both provide an important barrier and a first line of defence against, amongst others, toxic substances (allergens, irritants), pathogen invasion, and trauma. Whereas much is known about key innate and adaptive immune events in skin [1–3], relatively little is known in this respect about oral mucosa [4, 5].

Allergic and irritant contact dermatitis (ACD and ICD) are common pathological conditions arising in both the oral mucosa and skin and can be caused by an adverse reaction to chemicals [6–9]. The key biological events leading to skin sensitization and elicitation of ACD as well as the more general inflammatory events underlying ICD are well documented [6, 10–12]. ICD involves an innate immune response whereas sensitization and ACD-reactions involve an innate immune response which triggers an adaptive immune response. Both are caused by chemicals penetrating the outermost layer of the skin (stratum corneum) in order to reach the underlying viable epidermal cells. Keratinocytes (KC) are subsequently activated and release danger signals in the form of proinflammatory cytokines (e.g., TNF*α*, IL-1*α*, IL-18, and CCL27) [3, 6, 13–17]. These proinflammatory cytokines diffuse into the underlying dermis where they in turn stimulate fibroblasts to secrete inflammatory mediators. Cytokines such as IL-6 and CXCL8 create a general inflammatory response stimulating the dermal infiltration of, for example, T cells, monocytes, macrophages, and neutrophils. Importantly, a chemotactic gradient is created which will enable allergen-exposed, maturing, CXCR4^hi^ LC to migrate from the epidermis towards CXCL12 in the dermis, whereas irritant-exposed, nonmaturing, CXCR4^lo^ LC will migrate towards CCL2/CCL5 in the dermis [18, 19]. Replenishment of immature LC into the skin is further regulated by CCL5 and CCL20 [20]. Thus, extensive cross talk between skin KC and fibroblasts and the differential chemokine receptor expression on maturing versus immature LC enable (a) LC migration from the epidermis into the dermis after allergen or irritant exposure and (b) subsequent replenishment of LC through recruitment of precursors to the epidermis [21].

Upon chemical allergen exposure, LC take up haptens, possibly complexed with proteins, and migrate via the dermis to the draining lymph nodes where they can initiate an adaptive immune response by presenting the hapten to T cells. Upon a second exposure to the same allergen, an adverse outcome in the form of ACD may be elicited [12]. If the chemical is an irritant however, LC migrating into the dermis can undergo a phenotypic change into a CD14/CD68 macrophage-like cell and remain in the dermis [22, 23]. Clearly skin KC and fibroblasts play key regulatory roles in the innate immune events of both ACD and ICD. In the oral cavity, allergen and irritant mediated inflammation can also occur and is known as contact stomatitis. Contact stomatitis can be caused by exposure to chemicals in toothpaste and mouth wash (e.g., the sensitizer cinnamaldehyde or the irritant SDS) or in dental medical devices (e.g., metals: palladium, nickel, and gold) [8, 24, 25]. However, very little is known about the innate immunological events that trigger these oral diseases. For instance, the cytokines and chemokines pivotal for LC activation and migration in oral mucosa are currently unknown. Evidence suggests that skin and oral mucosal tissues react differently to environmental insults. For example, CCL27, a general (pro)inflammatory chemokine, is predominantly secreted by skin KC [26] whereas its homologue CCL28 is predominantly secreted by mucosa KC [27, 28]. Both share the same receptor CCR10 which mediates T cell homing whereas CCL28 also binds to CCR3, which can mediate eosinophil and Th1 and Th2 recruitment [29]. Whereas CCL27 is implicated in inflammatory skin disease such as psoriasis [15], CCL28 has been shown to mediate migration of antibody secreting plasma cells to mucosal tissues [27, 28].

Notably, very clear differences are also found in the histology between skin and oral mucosa, of the epithelial tissue in particular. Gingiva epithelium demonstrates a highly proliferative, thicker epithelium compared to skin epithelium and also has different epithelial localization of keratins, SKALP, loricrin, and involucrin [29, 30]. With such distinct (immuno)histological differences between the two tissues, it can be expected that differences also exist between their innate responses to environment assault. The study in three-dimensional tissue explants of the differential regulation of innate immune responses and LC migration in homeostasis and disease between skin and oral mucosa is complicated by the scarcity of available oral mucosa samples as these are generally infected or inflamed and are only available as very small pieces (3–6 mm diameter biopsies). This means that extensive studies using excised oral mucosa cannot be carried out as we have done in the past, for example, the study of LC migration in skin [18–20, 23]. Furthermore, the use of intact biopsies, which contain many different cell types, makes it difficult to dissect specific cross talk between particular cell types.

In this study, in order to overcome the abovementioned limitations, we have employed tissue engineered oral mucosa and skin equivalents, consisting of a fully differentiated epithelium on a fibroblast-populated connective tissue matrix, to carry out comparative analyses of skin and oral gingival-derived cytokines and chemokines involved in LC migration, both in the steady state and after stimulation with proinflammatory cytokine TNF*α*, or upon topical exposure to a known allergen and irritant [17, 30–32].

## 2. Materials and Methods

### 2.1. Cell Culture

Human adult skin and gingiva were obtained after informed consent from patients undergoing abdominal dermolipectomy or wisdom tooth extraction, respectively, and used in an anonymous fashion in accordance with the “Code for Proper Use of Human Tissues” as formulated by the Dutch Federation of Medical Scientific Organizations (http://www.fmwv.nl/), following procedures approved by the Institutional Review Board of the VU University Medical Center. Skin and gingiva samples were not donor matched.

*Epithelial KC*. Adult skin and gingiva KC, isolated from 3–6 mm punch biopsies, were cultured under similar conditions essentially as described earlier [33]. KC were cultured at 37°C, 7.5% CO_2_ in KC medium consisting of Dulbecco's Modified Eagle Medium (DMEM) (Lonza, Basel, Switzerland)/Ham's F-12 (Gibco, Grand Island, USA) (3 : 1), 1% Ultroser G (BioSepra S.A. Cergy-Saint-Christophe, France), 1% penicillin-streptomycin (Gibco), 1 *μ*mol/L hydrocortisone, 1 *μ*mol/L isoproterenol, and 0.1 *μ*mol/L insulin and containing 1 ng/mL keratinocyte growth factor (KGF) for skin keratinocytes or epidermal growth factor (EGF) for gingival keratinocytes. Keratinocytes grew in colonies of proliferating and differentiating cells and were passaged when 90% confluent, using 0.5 mM EDTA/0.05% trypsin (Gibco), and used for experiments at passage 2. Importantly, the KC were kept in culture for the same period of time (10–12 days) to eliminate confounding culture aging effects.

*Fibroblasts*. Adult skin and gingiva fibroblasts were isolated and cultured under identical conditions. In short, fibroblasts were enzymatically isolated from 3–6 mm punch biopsies and were cultured in DMEM containing 1% Ultroser G, 1% penicillin-streptomycin at 37°C, and 5% CO_2_ essentially as described previously [34]. Cultures were passaged when 90% confluent and used for experiments at passage 3. These fibroblast cultures are >99% CD90 positive (flow cytometry). Importantly, the skin and gingiva fibroblasts were cultured for the same period of time (28–35 days) to eliminate confounding culture aging effects.

*Skin Equivalent (SE) and Gingiva Equivalent (GE) Culture*. Reconstruction of SE and GE was achieved by seeding KC (0.5 × 10^6^ cells) onto fibroblast-populated collagen gels as previously described [32]. Cells were submerged for 3 days in KC medium (see above) containing 1 ng/mL EGF. To induce epithelial differentiation, the constructs were lifted to air-liquid interface and cultured for 14 days in KC medium containing 0.2% Ultroser G, 1 × 10^−5 ^M L-carnitine, 1 × 10^−2 ^M L-serine, 50 *μ*g/mL ascorbic acid, and 2 ng/mL EGF. Unless otherwise stated, all additives were purchased from Sigma Chemical Co. (St. Louis, MO, USA).

*Chemical Exposure*. Finn Chamber filter paper discs of 11 mm diameter (Epitest, Oy, Finland) for SE or 03-150/38 gauze filters of 12 mm diameter (Sefar Nitex, Heiden, Switzerland) for GE were impregnated with the allergen cinnamaldehyde (CA) in 1% DMSO in water v/v or irritant SDS in water (Sigma Chemical Co.). The chemicals or vehicle (control) impregnated discs or gauzes were applied topically to the cultures for 24 h at 37°C, 7.5% CO_2_ at nontoxic concentrations (>70% viability). Viability was determined by MTT assay as described previously [16].

### 2.2. Cytokine Exposure Experiments

*Cell Monolayer Exposure to rhTNFα or rhIL-1α*. Subconfluent fibroblast and KC monolayer cultures grown in 6-well plates were exposed to serial dilutions of rhTNF*α* or rhIL-1*α* (0, 100, or 200 International Units/mL) (Strathmann Biotech, Hamburg, Germany) for 4 h in 1.5 mL medium, after which the cells were washed with PBS and fresh culture medium (see above) was added. After 24 h the culture supernatant was harvested and stored at −20°C for ELISA analysis.

*Tissue Equivalent Exposure to rhTNFα*. SE and GE cultures were exposed to serial dilutions of rhTNF*α* or rhIL-1*α* (0, 100, or 200 International Units/mL) for 24 h in 1.5 mL medium. After 24 h the culture supernatant was harvested and stored at −20°C for ELISA analysis.

### 2.3. Immunohistochemical Staining

All procedures for paraffin embedded sections and immunohistochemical staining were performed as previously described [30, 31]. Primary monoclonal antibodies directed against keratin 10 (clone DE K10, ICN Biomedicals, Zoetermeer, The Netherlands) and loricrin (AF 62, BioLegend, San Diego, CA, USA) were used. All sections were counterstained with Mayer's haematoxylin. Negative controls were prepared by substituting the primary antibody with an isotype control antibody. For morphological analysis, haematoxylin and eosin staining was used. The sections were embedded in Aquatex.

### 2.4. ELISA

Duoset CCL2, CCL5, CCL20, CCL27, CCL28, IL-18, or CXCL12 development systems (R&D Systems), PeliKine compact kits IL-1*α*, IL-1*β*, IL-6, or CXCL8 development systems (Sanquin, Amsterdam, The Netherlands), and a TNF*α* high sensitive detection set were all used as described by the suppliers (R&D Systems).

### 2.5. Statistical Analysis

Statistical significance of differences between the unexposed and exposed equivalents, KC, or fibroblasts was calculated using a paired *t*-test. For comparisons between skin and gingiva, an unpaired *t*-test was used. Both tests used 2-way ANOVA followed by Dunnett's multiple comparison using GraphPad version 6.0.

## 3. Results

### 3.1. Tissue Engineered Full Thickness Skin and Gingiva Equivalents

**Figure 1:**
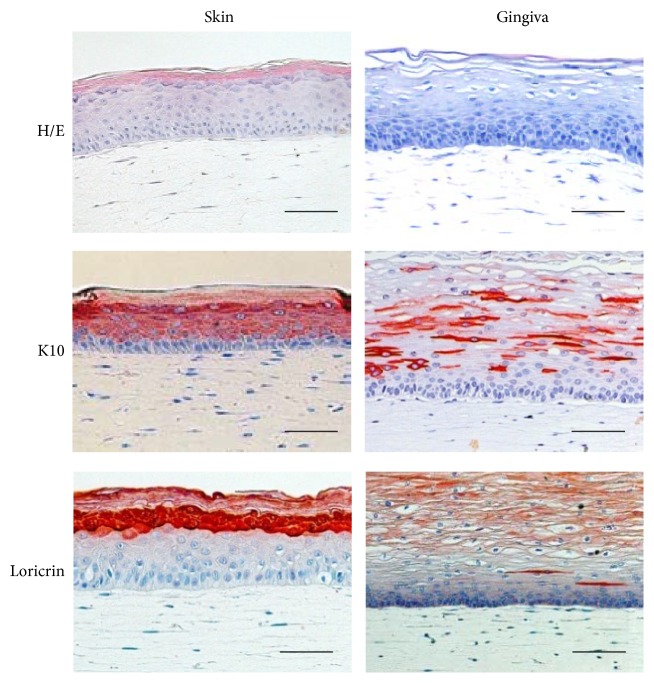
Full thickness tissue engineered skin and gingiva. Haematoxylin and eosin (H/E) staining and immunohistochemical analysis with keratin 10 (K10) and loricrin in tissue sections illustrate the different representative characteristics of skin and gingiva equivalents. Scale bar: 50 *μ*m.

In order to be able to study cytokine secretion in human skin and gingiva in a physiologically relevant model, full thickness skin and gingiva equivalents were constructed (Figure 1). Both skin and gingiva models consisted of a reconstructed epithelium (skin- or gingiva-derived KC) on a fibroblast- (skin- or gingiva-derived) populated collagen hydrogel, which served as the connective tissue matrix. The characteristic intrinsic properties of skin and gingiva are illustrated by the phenotypic differences observed in the epithelium. The SE epidermis consisted of a compact basal layer of KC, a spinous layer, a stratum granulosum, and a stratum corneum. Keratin 10 was strongly expressed in all suprabasal KC and loricrin was strongly expressed only in the stratum granulosum. In contrast, the GE epithelium showed a thicker multilayer of KC with increasing differentiation (flattening) of the KC away from the basal layer. The epithelium lacked a clearly defined stratum granulosum and a stratum corneum. Keratin 10 and loricrin were only intermittently expressed in the suprabasal KC. These results correlated closely to our previous published results for native skin and gingiva [30].

### 3.2. Basal Cytokine Secretion by Skin and Gingiva Keratinocytes and Fibroblasts

**Figure 2:**
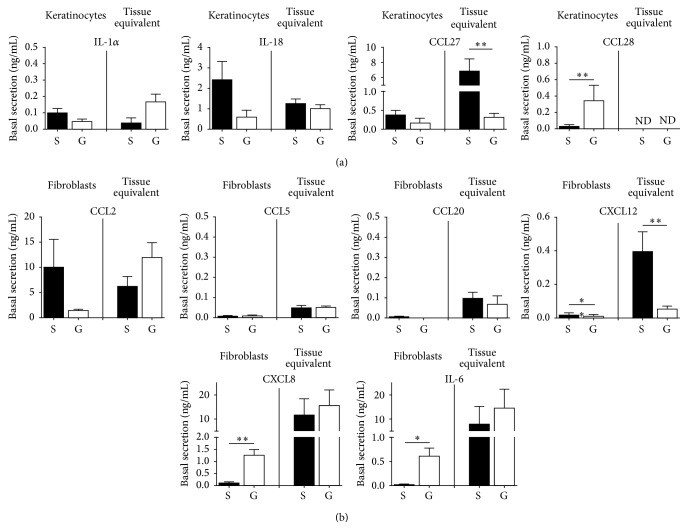
Basal cytokine and chemokine secretion by keratinocytes, fibroblasts, and tissue equivalents. (a) Cytokine/chemokine secretion by skin (S) and gingiva (G) keratinocytes and tissue equivalents. (b) Cytokine/chemokine secretion by skin and gingiva fibroblasts and tissue equivalents. Culture supernatants were collected over 24 h and analysed by ELISA. Data represent the average of at least 8 individual experiments ± SEM; ^*∗*^ *p* < 0.05; ^*∗∗*^ *p* < 0.01 (unpaired Student's *t*-test). ND = not detectable.

Homeostatic migration of immune cells including LC and their progenitors is regulated by basal secretion of cytokines and chemokines. Therefore, we first investigated basal secretion of proinflammatory mediators secreted by epithelial KC and tissue equivalents derived from skin and gingiva (Figure 2(a)). Skin and gingiva showed similar levels of secretion of IL-1*α* and IL-18. However, CCL27 secretion was more than 20-fold greater in SE compared to GE (with a similar trend for KC cultures), and CCL28 secretion was more than 10-fold greater in gingiva KC compared to skin KC. CCL28 secretion was below the detection limit of the ELISA in both SE and GE cultures.

Next we determined the basal secretion of inflammatory chemokines by skin and gingiva fibroblasts and tissue equivalents (Figure 2(b)). Notably the chemokine CXCL12 which is pivotal in mediating epidermis-to-dermis migration of maturing LC [18] was clearly secreted at greater levels by skin fibroblasts and SE as compared to gingiva fibroblasts and GE (more than a 7-fold difference between the tissue equivalents), whereas other chemokines involved in irritant induced immature LC migration (CCL2, CCL5 [19]) and LC precursor immigration to the epidermis (CCL20 [20]) were secreted at similar levels by skin and gingiva. In contrast, the general inflammatory cytokines CXCL8 and IL-6 were secreted in larger amounts by gingiva fibroblasts than by skin fibroblasts (15-fold and 3-fold, resp.) although this difference was no longer apparent when comparing the tissue equivalents. Cytokines IL-1*β* and TNF*α* were not detectable in any of the culture models. These results indicate that skin and gingiva cells have different intrinsic capacities for basal steady-state secretion of (pro)inflammatory cytokines.

### 3.3. Differential Cytokine Secretion by Skin and Gingival Keratinocytes and Fibroblasts in Response to TNF*α*

**Figure 3:**
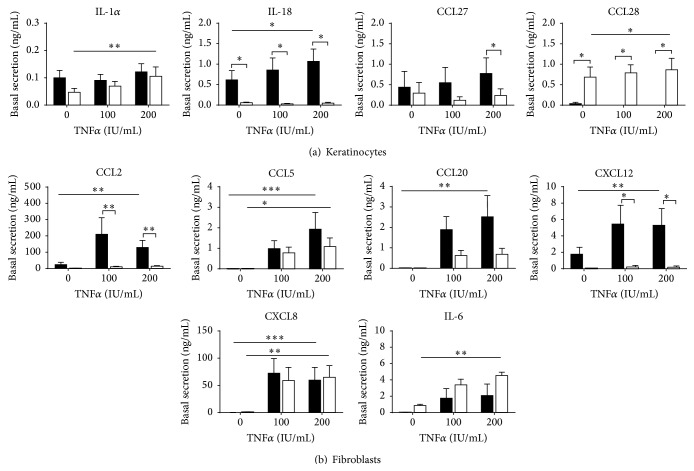
Differential cytokine and chemokine secretion by keratinocytes and fibroblasts in response to rhTNF*α*. (a) Skin compared to gingiva keratinocytes; (b) skin compared to gingiva fibroblasts. Cultures were unexposed or exposed to rhTNF*α* (100 and 200 IU/mL) for 24 h in the culture medium. Culture supernatants were collected and analysed by ELISA. Data represent the average of at least 3 individual experiments ± SEM; ^*∗*^ *p* < 0.05; ^*∗∗*^ *p* < 0.01; paired Student's *t*-test for intraskin and intragingiva comparisons, unpaired Student's *t*-test for skin versus gingival comparisons.

TNF*α* is a major proinflammatory cytokine described to initiate LC migration and inflammatory responses [35]. Therefore, we next determined how rhTNF*α* influenced the (pro)inflammatory cytokine secretion by skin- and gingival-derived KC and fibroblasts. Supplementation of KC culture medium with rhTNF*α* only slightly increased secretion of IL-18 by skin KC and IL-1*α* and CCL28 by gingiva KC. Secretion of IL-18 and CCL27 remained higher from skin KC, whereas CCL28 secretion remained higher from gingiva KC (Figure 3(a)).

In contrast to KC, rhTNF*α* clearly caused a dose dependent increase in secretion of all six cytokines (CCL2, CCL5, CCL20, CXCL12, CXCL8, and IL-6) from both skin- and gingiva-derived fibroblasts (Figure 3(b)). Notably, in response to rhTNF*α*, three of the four chemokines reported to play a role in LC migration in skin were secreted more abundantly by skin fibroblasts than by gingiva fibroblasts (CCL2, CCL20, and CXCL12 but not CCL5). In contrast, the general inflammatory cytokines CXCL8 and IL-6 were secreted in similar amounts by both skin and gingiva fibroblasts in response to rhTNF*α*. Similar results were obtained when cultures were supplemented with rhIL-1-*α* (data not shown).

Taken together, these results clearly show differential proinflammatory cytokine-induced secretion of keratinocyte- (IL-18, CCL27, and CCL28) and fibroblast-derived chemokines (CCL2, CCL20, and CXCL12) between skin and gingiva.

### 3.4. Differential Cytokine Secretion by Skin and Gingiva Tissue Equivalents in Response to TNF*α*, Allergen, and Irritant

**Figure 4:**
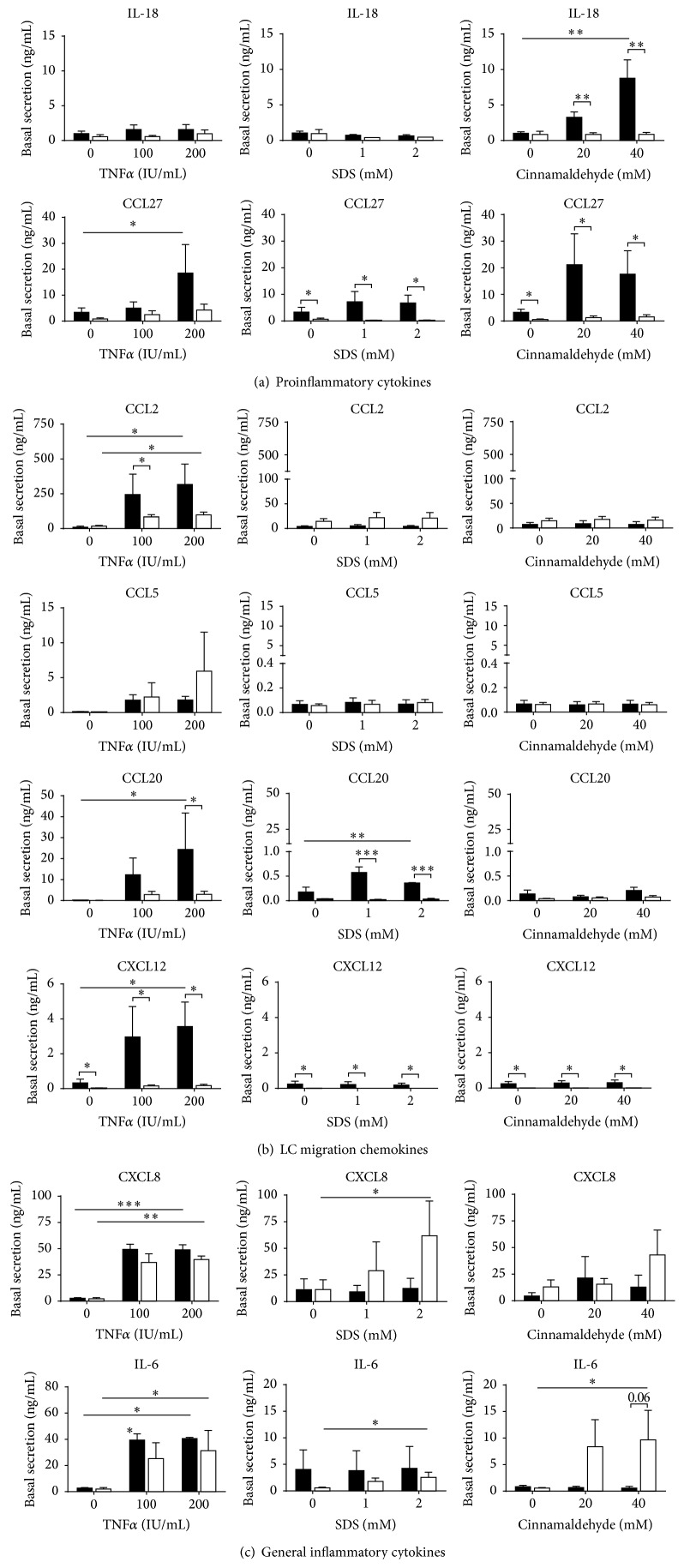
Differential cytokine and chemokine secretion by skin and gingiva equivalents in response to rhTNF*α*, irritant SDS, and allergenic cinnamaldehyde. (a) Proinflammatory cytokine secretion; (b) Langerhans cell (LC) migration related chemokine secretion; (c) general inflammatory cytokine secretion. Cultures were unexposed or exposed to rhTNF*α* (culture medium exposure; 100 and 200 IU/mL), SDS (topical exposure; 1 and 2 mM), or cinnamaldehyde (topical exposure; 20 and 40 mM) for 24 h. Cytotoxicity as assessed by MTT assay was <20% at the highest chemical concentration. Culture supernatants were collected and analysed by ELISA. Data represent the average of at least 3 individual experiments ± SEM; ^*∗*^ *p* < 0.05; ^*∗∗*^ *p* < 0.01; ^*∗∗∗*^ *p* < 0.001; paired Student's *t*-test for intraskin and intragingiva comparisons, unpaired Student's *t*-test for skin versus gingival comparisons.

Upon topical exposure of the skin to a chemical irritant or allergen, a rhTNF*α* mediated innate inflammatory response is initiated [17]. In order to investigate this in more detail and importantly to determine similarities and differences between skin and gingiva in the context of cellular cross talk in a 3D tissue environment, tissue engineered full thickness equivalents were exposed to rhTNF*α* (in culture medium), the irritant SDS (topical), or the allergen cinnamaldehyde (CA; topical) (Figure 4; Table 1). Notable differences were observed in the keratinocyte-derived proinflammatory cytokine and chemokine secretion profiles between skin and gingiva (Figure 4(a)). IL-18 showed a dose dependent increase in secretion only in response to the allergen CA in SE. This was not observed in GE, nor was it observed in SE or GE in response to rhTNF*α* or SDS. Chemokine CCL27, on the other hand, showed a dose dependent increase in secretion from SE in response to TNF*α*, SDS, and CA, but not from GE. IL-1*α* and CCL28 secretion were below the detection limit of the ELISA in all experimental conditions.

Next, fibroblast-derived chemokines described to play key roles in LC migration and recruitment were studied (Figure 4(b)). An increase in secretion of CCL2, CCL5, CCL20, and CXCL12 was observed in response to rhTNF*α* in both SE and GE. Notably CCL2, CCL20, and CXCL12 were secreted at higher levels by SE than by GE. Similarly, in response to noncytotoxic concentrations (>80% viable) of SDS, SE but not GE showed an increase in secretion of CCL20. Neither exposure to SDS nor exposure to CA resulted in an increase in secretion of CCL2, CCL5, or CXCL12 from the SE or GE. CXCL12 secretion (similarly to secretion of CCL20) was higher in SE as compared to GE. Cytokines involved in general inflammation (CXCL8, IL-6) showed a similar increase in secretion in response to rhTNF*α* in both SE and GE. However, secretion of these two cytokines was only increased by GE and not by SE when exposed to SDS or CA (Figure 4(c)). This increase resulted in a trend difference (*p* < 0.06) in IL-6 secretion between the two tissue types when exposed to CA.

Taken together, these results indicate that clear differences exist between skin and gingiva with regard to innate immune regulation by KC- and fibroblast-derived cytokines and chemokines. In particular, IL-18, CCL2, CCL20, and CXCL12, which mediate LC migration, are predominantly secreted by SE exposed to rhTNF*α*, the irritant SDS, or the allergen CA.

## 4. Discussion

In this study, we made an extensive comparison of oral mucosa and skin with regard to release of cytokines and chemokines involved in LC migration, as well as general inflammation. In order to do this, we used in addition to conventional KC and fibroblast cultures physiologically relevant 3D tissue engineered skin and gingiva equivalents. Environmental assault was mimicked by exposing cultures to the proinflammatory cytokine TNF*α* via the culture medium, or by topically exposing the epithelium to a chemical allergen (CA) and a chemical irritant (SDS). We found that key chemokines described to be responsible for LC migration in skin, that is, IL-18, CCL2, CCL20, and, most notably, CXCL12, were clearly secreted at higher levels by skin as compared to gingiva, suggesting that different and as yet unknown innate mechanisms are involved in mediating and controlling LC migration in gingiva.

Proinflammatory IL-18 together with IL-1*β* and TNF*α* is necessary for skin LC to migrate from the epidermis [13, 14]. IL-18 has been shown to play a vital and early role in the induction of allergic contact sensitization [13, 36]. Indeed, IL-18 is now an accepted biomarker in* in vitro* assays to identify and discriminate contact allergens from respiratory sensitizers and irritants [16, 37]. Here we show in line with these findings that IL-18 secretion is only increased in SE exposed to the allergen CA and is not increased by the irritant SDS. However, remarkably, we show that this cytokine is hardly secreted by gingiva in our study. In all our experimental conditions, a very low, noninducible amount was detected in gingiva culture supernatants compared to skin, suggesting that IL-18 is not required to mobilize LC in gingiva. Indeed endogenous IL-18 in experimentally induced asthma was found to be irrelevant for clinical symptoms, and therefore our finding that IL-18 may not be required to mobilize gingiva LC may possibly refer to other mucosal tissues as well [38]. In contrast to our results, it was previously shown that bioactive IL-18 was detected in the supernatant of human oral epithelial cells upon combined stimulation with neutrophil proteinase 3 (PR3) and LPS after IFN*γ*-priming [39]. This would suggest that IL-18 may be inducible by pathogenic but not by chemical allergen stimuli in the oral cavity.

Previously we have shown that CXCL12 is a key chemokine in mediating migration of maturing LC from epidermis to dermis [18], CCL2 and CCL5 are key chemokines in mediating LC migration after irritant exposure [19], and CCL5 and CCL20 are involved in LC replenishment in the epidermis [20]. Our finding that CCL2 and especially CXCL12 are predominantly secreted by skin as compared to gingiva in response to rhTNF*α* (and for CXCL12 also in response to chemical exposure) indicates a clear difference in mechanisms regulating LC migration from the epithelium to connective tissue in skin as compared to gingiva. This difference in chemokines regulating LC migration is further supported by the finding that CCL20 was also significantly higher in skin after rhTNF*α* and SDS exposure. Only CCL5 was secreted at similar levels in skin and gingiva. Although it is clear that oral LC do have the ability to migrate from the epithelium into the lamina propria upon environmental assault [4, 40, 41], the underlying mechanisms are still unknown and our results suggest that they may differ considerably from those found in skin.

The tissue engineered skin and gingiva equivalents, and in particular the reconstructed epithelium, used in this study, closely represented their native counterparts. Not only the histology but also keratin and loricrin expression mimicked the different types of epithelium in line with our previous studies [30, 31]. Our current results show that basal cytokine secretion is also different in these two tissues. CCL27 was predominantly secreted by skin KC whereas CCL28 was predominantly secreted by gingiva KC in line with studies using patient derived biopsies and biological samples [26–28, 42]. Notably, CCL28 was only detected in gingiva KC cultures and not in GE, even when stimulated with rhTNF*α*, indicating that CCL28 may possibly be directly involved in cross talk between gingiva keratinocytes and fibroblasts, with possible consumption by fibroblasts in the GE accounting for the observed lack of detectable levels therein. Indeed, previously we have shown that the skin homologue CCL27 has proinflammatory properties and that it can stimulate adipose derived stromal cells to secrete VEGF, CXCL1, CXCL8, and IL-6 [26].

Whereas differential secretion was observed for cytokines involved in LC migration, this was not the case for the general inflammatory mediators CXCL8 and IL-6 which were upregulated by rhTNF*α* in skin and gingiva to a similar extent. CA and SDS did however result in a dose dependent increase only in GE. This indicates that mechanisms controlling general inflammation are different from those controlling LC migration and require further investigation.

In conclusion, our results indicate that the cytokines and chemokines involved in triggering and mediating LC migration and the innate immune response are different in skin and gingiva. Since extensive cross talk between keratinocytes, fibroblasts, and LC may direct and control LC migration, in future studies physiologically relevant immune competent skin and gingiva models with integrated LC may be used to investigate this further in a fully defined and standardized manner [32, 43].

## Figures and Tables

**Table 1:** Summary of results obtained after exposing skin and gingiva tissue equivalents to TNF*α*, an allergen (CA), or an irritant (SDS).

Experimental condition	Dose response within tissue^a^		Dose response between tissues SE > GE^b^	2 comparisons between tissues^c^
	Skin	Gingiva		
Proinflammatory cytokines				
IL-18				
TNF*α*	ns	ns	ns	ns
SDS	ns	ns	ns	ns
CA	*∗∗*	ns	*∗∗*	^*∗∗*^SE > GE
CCL27				
TNF*α*	*∗*	ns	ns	ns
SDS	ns	ns	*∗*	^*∗*^SE > GE
CA	ns^d^	ns	*∗*	^*∗*^SE > GE

LC migration chemokines				
CCL2				
TNF*α*	*∗*	*∗*	ns	^*∗*^SE > GE
SDS	ns	ns	ns	ns
CA	ns	ns	ns	ns
CCL5				
TNF*α*	ns	ns	ns	ns
SDS	ns	ns	ns	ns
CA	ns	ns	ns	ns
CCL20				
TNF*α*	*∗*	ns	ns	^*∗*^SE > GE
SDS	*∗∗*	ns	*∗∗∗*	^*∗∗*^SE > GE
CA	ns	ns	ns	ns
CXCL12				
TNF*α*	*∗*	ns	*∗*	^*∗*^SE > GE
SDS	ns	ns	*∗*	^*∗*^SE > GE
CA	ns	ns	*∗*	^*∗*^SE > GE

General inflammatory mediators				
CXCL8				
TNF*α*	*∗∗∗*	*∗∗∗*	ns	ns
SDS	ns	*∗*	ns	ns
CA	**ns**	ns	ns	ns
IL-6				
TNF*α*	*∗∗*	*∗*	ns	ns
SDS	ns	*∗*	ns	ns
CA	ns	*∗*	ns	ns (0.06 GE > SE)

^a^Dose response within tissue: statistical significance of differences between the unexposed and exposed equivalents, KC, or fibroblasts was calculated using a paired *t*-test.

^b^Dose response between tissues: SE > GE. For comparisons between dose responses of skin and gingiva, an unpaired *t*-test was used.

^c^Two single comparisons between tissues (unpaired *t*-test).

^d^Skin/CCL27: significant difference between 0 and 20 mM CA (*p* < 0.05; paired *t*-test) but not in entire dose response.

Tests used 2-way ANOVA followed by Dunnett's multiple comparison using GraphPad version 6.0, Data represent the average of at least 3 individual experiments ± SEM ^*∗*^ *p* < 0.05; ^*∗∗*^ *p* < 0.01; ^*∗∗∗*^ *p* < 0.001.

## Abbreviations

CA: Cinnamaldehyde
GE: Gingiva equivalent
KC: Keratinocyte
SDS: Sodium dodecyl sulfate
SE: Skin equivalent.
